# Thermal Annealing Effect on Optical Properties of Binary TiO_2_-SiO_2_ Sol-Gel Coatings

**DOI:** 10.3390/ma6010076

**Published:** 2012-12-24

**Authors:** Xiaodong Wang, Guangming Wu, Bin Zhou, Jun Shen

**Affiliations:** Shanghai Key Laboratory of Special Artificial Microstructure Materials and Technology, Pohl Institute of Solid State Physics, Tongji University, Siping Road 1239, Shanghai 200092, China; E-Mails: xiaodong_wang@tongji.edu.cn (X.W.); wugm@tongji.edu.cn (G.W.); zhoubin863@tongji.edu.cn (B.Z.)

**Keywords:** binary TiO_2_-SiO_2_, sol-gel, optical coating, thermal annealing

## Abstract

TiO_2_-SiO_2_ binary coatings were deposited by a sol-gel dip-coating method using tetrabutyl titanate and tetraethyl orthosilicate as precursors. The structure and chemical composition of the coatings annealed at different temperatures were analyzed by Raman spectroscopy and Fourier Transform Infrared (FTIR) spectroscopy. The refractive indices of the coatings were calculated from the measured transmittance and reflectance spectra. An increase in refractive index with the high temperature thermal annealing process was observed. The Raman and FTIR results indicate that the refractive index variation is due to changes in the removal of the organic component, phase separation and the crystal structure of the binary coatings.

## 1. Introduction

In optical thin film application, it is important to have tunable refractive indices over a range as large as possible and continuously if feasible. Due to the low cost, wide spectral region of transparency from visible to near infrared [1], as well as the high chemical and thermal stabilities, SiO_2_ and TiO_2_ are commonly used as low and high refractive index materials, respectively. So far, they have been successfully used in optical device applications, such as antireflective coatings [2], high reflecting mirrors [3], beam splitter [4] and planar waveguides [5]. Binary TiO_2_-SiO_2_ coatings cover a wide range of achievable refractive index [6,7] from approximately 1.45 to 2.55 because of the large difference in refractive index of the two compounds. A large number of deposition techniques including electron beam evaporation [8], sputtering [9], pulsed laser deposition [10], sol-gel [11,12,13] and chemical vapor deposition [14] have been used to prepare the binary TiO_2_-SiO_2_ coatings. However, most reports deal with these types of thin films on an empirical basis, without correlating the actual values of the optical parameters with the type of thin film microstructure and the composition. The detailed influence of microstructure or that of a partial or total segregation of the pure oxides is still not available. A recent exception in this regard is the work of Louis *et al.* [12], which constitutes a thorough investigation of the correlations between the microstructure of SiO_2_/TiO_2_ thin films and their optical properties. They investigated how the binary coating is affected by the mixing ratio of the two oxides and the thermal annealing of the samples through an *in situ* thermal ellipsometry analysis. They showed that the silica proportion was critical to the crystallization of TiO_2_ as well as to the optical properties. However, as the binary coating is synthesized by a chemical sol-gel method, the detailed annealing influence on composition and microstructure with respect to their optical properties has neither been of concern nor has it been studied. It is of interest to study the thermal annealing effect on the change of optical properties of the binary coatings.

In this work, we report on the synthesis and characterization of binary TiO_2_-SiO_2_ optical coatings prepared by a sol-gel method. The synthesized coatings were normally thermally annealed to achieve certain optical and mechanical properties. The crystal structures and composition of the coatings annealed at various temperatures were investigated by Raman and FTIR spectroscopy, respectively. The refractive indices of the coatings were extracted from reflectance and transmittance spectra. The comprehensive analysis of the thermal annealing effect on the optical properties of binary TiO_2_-SiO_2_ coatings was then discussed. We demonstrated that the increase in refractive index was due to the combined effects of the removal of the organic component, phase separation and the crystal structure transition of the binary coatings.

## 2. Experimental Section

### 2.1. Sol Synthesis

Sol was prepared by dissolving tetrabutyl titanate (Ti(OC_4_H_9_)_4_, TBOT) and tetraethyl orthosilicate (Si(OC_2_H_5_)_4_, TEOS) in anhydrous ethanol (C_2_H_5_OH, EtOH). Deionized water (H_2_O) was used for hydrolysis, with acetylacetone (CH_3_COCH_2_COCH_3_, AcAc) as chelating agent and acetic acid (CH_3_COOH, HAc) as catalyst. During the synthesis, two different but equal parts of ethanolic solutions were prepared. In the first part, TBOT was dissolved in anhydrous ethanol containing AcAc. After mixed with HAc, the solution was then sealed and stirred for 30 min to achieve a complete chelation between the alkoxide and AcAc. The second part of the solution was then prepared by mixing the TEOS and deionized water with anhydrous ethanol. These two solutions were then mixed and stirred for 2 h to achieve hydrolysis and condensation. The molar ratio was TBOT:TEOS:EtOH:H_2_O:HAc:AcAc = 1:1:30:5:2:1. The mixture was finally aged in a stable environment (with humidity lower than 30% and temperature of 20~25 °C) for 48 h.

### 2.2. Coating Preparation

The silicon wafer and silica glass substrates were firstly cleaned thoroughly, heated at 200 °C for 20 min, and then cooled down to room temperature. A dip-coating apparatus (CHEMAT Dip Master-200) was used for the depositions, and the film thickness could be adjusted by the withdrawal rate (0~12 inch/min). After each coating, the films were first pretreated at 100 °C for 1h, and then heat-treated in a muffle furnace for 2 h at different temperatures ranging from 300 to 900 °C. All the coating processes of the samples were the same to make sure that the properties of the films annealed at different temperatures could be accurately compared and studied.

### 2.3. Characterization

Raman spectra were recorded at room temperature with a Jobin-Yvon micro-Raman apparatus (HR-800) equipped with a 20 mW Ar^+^ laser (Spectra-Physics, 163-C-42) emitting at 514 nm, and a microscope (Olympus BX41). An edge filter was used in the Raman setup to block the Rayleigh scattering light and stray laser bands. The laser beam irradiating the sample was attenuated to below 0.1 mW in order to avoid laser-induced heating. Before each measurement, a standard silicon wafer was used for the wavenumber calibration of the spectrometer. The spectral resolution was estimated to be 0.65 cm^−1^. Fourier transform infrared spectroscopy measurements were performed in the range of 400~4000 cm^−1^ using an FTIR spectrometer (BRUKER TENSOR 27). The transmittance and reflectance spectra of the films were measured in the 250~1000 nm region using a UV-VIS-NIR double beam spectrometer (JASCO-V570).

## 3. Results and Discussion

**Figure 1:**
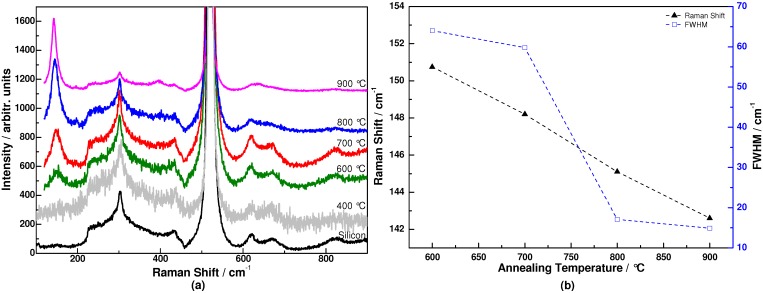
(**a**) Raman spectra of coatings annealed at different temperatures; (**b**) Peak position and full width at half maximum (FWHM) of E_g_ Raman peak.

Figure 1a shows the evolution of the Raman spectra of binary TiO_2_-SiO_2_ coatings after thermal annealing at different temperatures. The spectra of coatings annealed below 600 °C do not differ from that of the Si substrate, which is in agreement with an amorphous state and also with the fact that crystallization is more difficult in the presence of SiO_2_ [12]. Indeed several authors [15,16] previously reported that silicon atoms, if intimately mixed with titanium atoms, act as barriers to the diffusion of the Ti atoms which tends to delay nucleation-growth of TiO_2_ crystallites. After annealing at 600 °C, a small and broad band was detected around 151 cm^−1^. This band can be assigned to the low wavenumber anatase E_g_ mode [17], which indicates the beginning of TiO_2_ crystallization. With increasing annealing temperatures, the E_g_ line becomes better defined and shifts towards smaller wavenumbers. After annealing at 900 °C, the spectrum shows a well-defined E_g_ line at ~143 cm^−1^, as well as two other characteristic lines [18] (197, 394 cm^−1^) of the TiO_2_ anatase phase. However, no rutile phase related Raman mode was detected. The presence of silica seems to not only delay the crystallization into anatase but also the transformation into rutile, which is likely due to the contribution of surface free energy associated with smaller crystallites, as recently shown by Smitha et al [15]. Absence of phase transition also tends to prove that TiO_2_ and SiO_2_ are homogeneously mixed at the molecular level.

The peak position and full width at half maximum (FWHM) of the E_g_ band during annealing are shown in Figure 1b. As can be seen from the figure, the peak position of anatase TiO_2_ E_g_ mode shifts from 151 to 143 cm^−1^ whereas the FWHM decreases from 64 to 14.9 cm^−1^ consecutively. The possible origins of the peak shift and sharpening are due to size-induced phonon confinement [19], non-stoichiometry and stress effect. The decrease in the crystallite dimension to the nanometer scale can cause wavenumber shift and broadening of the Raman bands as a result of phonon confinement. During annealing, the increase of annealing temperature improves the crystalline quality of TiO_2_ and also causes an increase of the TiO_2_ grain size. The increasing crystal size reduces the phonon confinement effect, whereas the improved crystalline quality decreases the FWHM. In addition, the phase separation of TiO_2_ and SiO_2_ relaxes the compressive stress effect of the SiO_2_ matrix on the TiO_2_ nano-grains, resulting in the E_g_ mode shifts toward lower wavenumbers. Therefore, all these effects need to be taken into consideration to explain the change of the E_g_ Raman mode.

**Figure 2:**
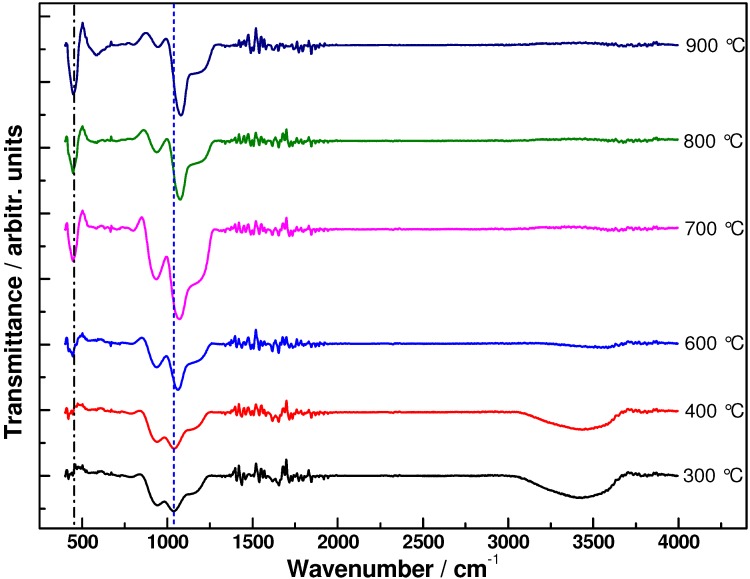
Fourier Transform Infrared spectra of coatings annealed at different temperatures.

To further understand more about the structural variation of the binary coatings during thermal annealing, the FTIR spectra of TiO_2_-SiO_2_ binary coatings annealed at different temperatures were performed and are shown in Figure 2. The strongest absorption peak approximately 1060 cm^−1^ is assigned to the asymmetric vibration of Si–O–Si bonds [20]. The absorption band approximately 900~1000 cm^−1^ is attributed to the stretching vibrations of Si–OH, SiO^−^ groups and Si–O–Ti bonds. The broad band approximately 3200~3600 cm^−1^ is attributed to the OH stretching vibration of different Si–OH species and to molecular water as well, and the band at 1616 cm^−1^ is attributed to adsorbed water. Both the bands decrease in intensity with increasing annealing temperature, showing that the hydroxyl groups are removed gradually from the coatings. Upon annealing at 600 °C, the intensity of the absorption band corresponding to Si–O–Si increases whereas the band approximately 900~1000 cm^−1^ decreases, which indicates the phase separation of crystalline TiO_2_ from the amorphous TiO_2_-SiO_2_ matrix.

**Figure 3:**
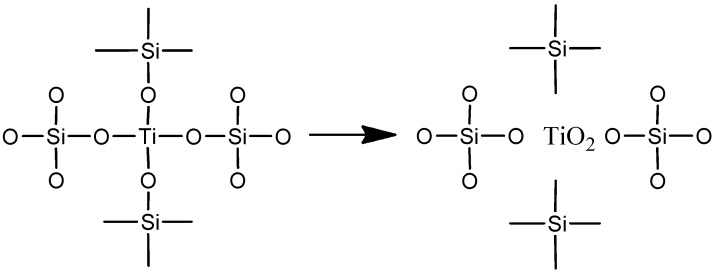
Schematic diagram of TiO_2_-SiO_2_ phase separation.

The phase separation process can be expressed as shown in Figure 3. With further increasing annealing temperatures, the band of anatase TiO_2_ (~445 cm^−1^) becomes more significant due to more complete crystallization of the TiO_2_ phase. In addition, the band approximately 1060 cm^−1^ can be clearly observed to shift towards higher wavenumber with increasing temperature, from approximately 1037 cm^−1^ after annealing at 300 °C to 1078 cm^−1^ after annealing at 900 °C. This position variation could be related to the elimination of oxygen deficiency [21]. After higher temperature treatment, TiO_2_-SiO_2_ phase separation together with TiO_2_ crystallization decrease the oxygen deficiency of SiO_2_, thereby resulting in a blue shift of the Si–O–Si vibration peak.

**Figure 4:**
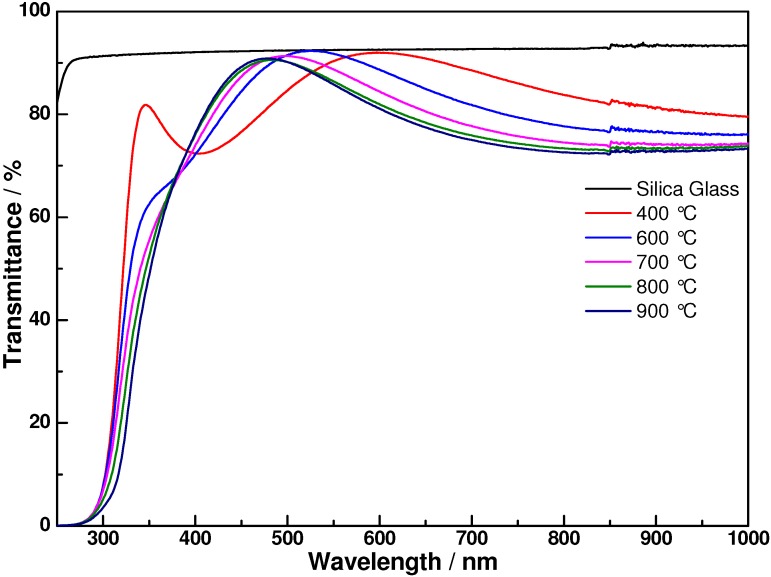
Transmittance spectra of SiO_2_-TiO_2_ coatings annealed at different temperatures.

Transmittance spectra of the coatings annealed at various temperatures in the wavelength region of 250 to 1000 nm are presented in Figure 4. Silica glasses were used as the substrate in these experiments to avoid the influence of the absorption edge of the substrate. As shown in Figure 4, the average transmittance of 400 °C annealed films is about 80% in the visible region with respect to silica glass substrate. It also can be seen clearly that the films are fully transparent in the visible region and start absorbing between 320 and 340 nm. Film annealed at 400 °C has the lowest absorption edge because the amorphous film has a large band gap. With increasing annealing temperature, it shows a gradual decrease in transmittance and the absorption edge shifts toward longer wavelength, indicating a decrease in the band gap of the films. This can be attributed to the structure change and the crystallization of the TiO_2_ content of the binary coatings.

The optical constants, e.g., refractive index, thickness, are a topic of fundamental and technological importance in optical coatings. In order to see the annealing effect on the optical properties of the coatings, the refractive indices were determined by the characterization technique based on least-square fitting to the measured reflectance and transmittance spectra [22]. For sol-gel dip-coating technology, both sides of the glass substrates were coated. Therefore, we built a five-layer (Air/coating layer/Silica glass/coating layer/Air) model to determine the optical constants of the film by the spectroscopic method. The Cauchy formula [23], which is an optical model for insulators and dielectric films, was used to describe the dispersion relationship of the film layer. The optical parameters of the coating layer were then determined from the measured transmittance and reflectance spectra for each coating by fitting simultaneously the results obtained from calculations and measurements over the 400~1000 nm region.

**Figure 5:**
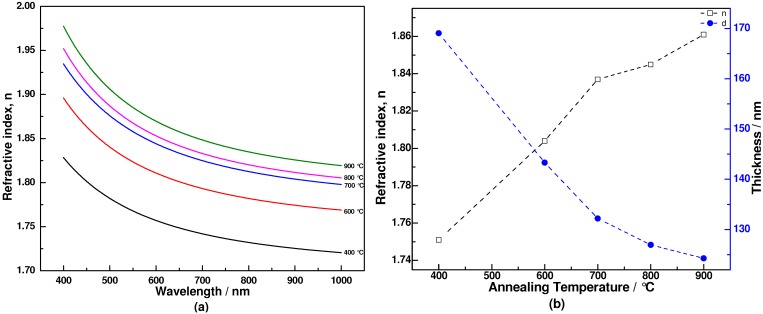
(**a**) Refractive index dispersion of binary coatings annealed at various temperatures; (**b**) Variation of refractive index (at 633 nm) and thickness of coatings annealed at various temperatures.

The determined refractive indices, as a function of wavelength for binary coatings annealed at different temperatures, are shown in Figure 5. Decreasing refractive index with wavelength indicates normal dispersion behavior. The data show that the refractive index increases with annealing temperature, from 1.75 to 1.86 at He-Ne laser wavelength of 633 nm. It is known that removal of the residual organic material and densification of the coating occurs during the thermal annealing process. Combined with the FTIR and Raman analysis, we know that the 400 °C annealed coating still has residual organic materials, and that the coating is composed of Ti–O–Si, amorphous SiO_2_ and TiO_2_, therefore resulting in a relatively low refractive index. After annealing at 600 °C, the removed organic groups, phase separation and the formed anatase TiO_2_ rapidly increase the refractive index of the binary coating. Further increase of the annealing temperature promotes the extent of crystallization and the thermal densification of the coating, thus increasing the refractive index slightly. Therefore, the increase of refractive index is due to complex changes in the organic component removal and changes caused by the film component, phase separation and crystal structure of the binary coatings. On the other hand, on removal of organic components and thermal densification, the binary coating thickness decreases continuously from 169 to 124 nm during the annealing process.

## 4. Conclusions

We studied the effect of thermal annealing on the microstructure and optical properties of binary TiO_2_-SiO_2_ coatings annealed at different temperatures. The results show that a rise in the heat-treatment temperature leads to a decrease in the amount of organic component as well as a structural reconstruction of the TiO_2_-SiO_2_ network together with TiO_2_ crystallization. These combined effects result in an increase of refractive index and a decrease of coating thickness. The present study will help to establish a better understanding of how the annealing process affects the optical properties of binary TiO_2_-SiO_2_ coatings.

## References

[1] Pal M., Pal U., Jiménez J.M.G.Y., Pérez-Rodríguez F. (2012). Effects of crystallization and dopant concentration on the emission behavior of TiO_2_:Eu nanophosphors. Nanoscale Res. Lett..

[2] Wang X., Shen J. (2010). Sol-gel derived durable antireflective coating for solar glass. J. Sol-Gel Sci. Technol..

[3] Zhang J., Chen X., Wang Z. (2010). High-damage-threshold broadband chirped mirror. Chin. Opt. Lett..

[4] Chen S.-H., Wang C.-H., Chai K.-Y., Chang T.-H., Yeh Y.-W., Lee C.-C., Ku S.-L., Huang C.-C. Polarization beam splitters with autocloned symmetric structure. Proceeding of Optical Interference Coatings.

[5] Que W., Zhou Y., Lam Y.L., Chan Y.C., Cheng S.D., Li H.P., Liu J., Kam C.H. (1999). TiO_2_/SiO_2_/ORMOSIL hybrid material planar waveguides prepared at low-temperature by sol-gel processing. Proc. SPIE.

[6] Yang L.L., Lai Y.S., Chen J.S., Tsai P.H., Chen C.L., Chang C.J. (2005). Compositional tailored sol-gel SiO_2_-TiO_2_ thin films: Crystallization, chemical bonding configuration, and optical properties. J. Mater. Res..

[7] Gracia F., Yubero F., Holgado J.P., Espinos J.P., Gonzalez-Elipe A.R., Girardeau T. (2006). SiO_2_/TiO_2_ thin films with variable refractive index prepared by ion beam induced and plasma enhanced chemical vapor deposition. Thin Solid Films.

[8] Sankur H., Gunning W. (1989). Crystallization and diffusion in composite TiO_2_-SiO_2_ thin films. J. Appl. Phys..

[9] Liu Y.Y., Qian L.Q., Guo C., Jia X., Wang J.W., Tang W.H. (2009). Natural superhydrophilic TiO_2_/SiO_2_ composite thin films deposited by radio frequency magnetron sputtering. J. Alloy. Compd..

[10] Sarkar D.K., Desbiens E., Khakani M.A.E. (2002). High-k titanium silicate dielectric thin films grown by pulsed-laser deposition. Appl. Phys. Lett..

[11] Nilchi A., Janitabar-Darzi S., Mahjoub A.R., Rasouli-Garmarodi S. (2010). New TiO_2_/SiO_2_ nanocomposites-phase transformations and photocatalytic studies. Colloids Surf. A.

[12] Louis B., Krins N., Faustini M., Grosso D. (2011). Understanding crystallization of anatase into binary SiO_2_/TiO_2_ sol-gel optical thin films: An *in situ* thermal ellipsometry analysis. J. Phys. Chem. C.

[13] Song C.F., Lv M.K., Yang P., Xu D., Yuan D.R. (2002). Structure and photoluminescence properties of sol-gel TiO_2_-SiO_2_ films. Thin Solid Films.

[14] Hodroj A., Chaix-Pluchery O., Audier M., Gottlieb U., Deschanvres J.L. (2008). Thermal annealing of amorphous Ti–Si–O thin films. J. Mater. Res..

[15] Smitha V., Manjumol K., Baiju K., Ghosh S., Perumal P., Warrier K. (2010). Sol-gel route to synthesize titania-silica nano precursors for photoactive particulates and coatings. J. Sol-Gel Sci. Technol..

[16] Machida M., Norimoto K., Watanabe T., Hashimoto K., Fujishima A. (1999). The effect of SiO_2_ addition in super-hydrophilic property of TiO_2_ photocatalyst. J. Mater. Sci..

[17] Wang X., Shen J., Pan Q. (2011). Raman spectroscopy of sol-gel derived titanium oxide thin films. J. Raman Spectrosc..

[18] Pärna R., Joost U., Nõmmiste E., Käämbre T., Kikas A., Kuusik I., Kink I., Hirsimäki M., Kisand V. (2012). Effect of different annealing temperatures and SiO_2_/Si(100) substrate on the properties of nickel containing titania thin sol-gel films. Phys. Status Solidi A.

[19] Bersani D., Lottici P.P., Ding X.-Z. (1998). Phonon confinement effects in the raman scattering by TiO_2_ nanocrystals. Appl. Phys. Lett..

[20] Darmawan A., Smart S., Julbe A., Diniz da Costa J.C. (2011). Iron oxide silica derived from sol-gel synthesis. Materials.

[21] Ono H., Ikarashi T., Ando K., Kitano T. (1998). Infrared studies of transition layers at SiO_2_/Si interface. J. Appl. Phys..

[22] Janicki V., Sancho-Parramon J., Stenzel O., Lappschies M., Görtz B., Rickers C., Polenzky C., Richter U. (2007). Optical characterization of hybrid antireflective coatings using spectrophotometric and ellipsometric measurements. Appl. Opt..

[23] Jenkins F.A., White H.E. (1981). Fundamentals of Optics.

